# The need to map existing health care services for refugees in Malaysia

**DOI:** 10.7189/jogh.11.03024

**Published:** 2021-01-30

**Authors:** Raudah Mohd Yunus, Nasibah Azme, Xin Wee Chen, Siti Fatimah Badlishah-Sham, Hayatul Najaa Miptah, Awla Mohd Azraai

**Affiliations:** 1 Department of Public Health Medicine, Faculty of Medicine, Universiti Teknologi MARA (UiTM), Sungai Buloh Campus, Malaysia; 2 Department of Physiology, Faculty of Medicine, Universiti Teknologi MARA (UiTM), Sungai Buloh Campus, Malaysia; 3 Department of Primary Care Medicine, Faculty of Medicine, Universiti Teknologi MARA (UiTM), Sungai Buloh Campus, Malaysia; 4 Department of Pathology, Faculty of Medicine, Universiti Teknologi MARA (UiTM), Sungai Buloh Campus, Malaysia

Despite hosting the largest number of refugees and asylum-seekers (RAS) compared to most other Southeast Asian countries [1], Malaysia is non-signatory to the 1951 UN Refugee Convention and lacks a clear socio-legal framework pertaining to RAS. To this date, there are close to 180 000 registered RAS in the country [2]. The vast majority are socio-economically marginalized and have restricted access to health care services, education and livelihoods. A survey by the UNHCR in 2015 reported that 23.9% of adult refugees in Malaysia had hypertension, 8% had diabetes, and 7% had cardiovascular diseases [3,4] Another study among Rohingya children in Kuala Lumpur found 27.5% to be underweight and 11.5% stunted [5]. These however are likely conservative estimates, given the hard-to-reach nature of RAS and other existing health issues that have not been adequately studied, like mental health.

The Malaysian government – through its Ministry of Health – provides a 50% subsidy (from the foreigner’s rate) to RAS at government clinics and hospitals. Nevertheless, medical expenses incurred following such reduction remain exorbitant, due to the continuous increase in fees and the current law that prohibits this group from formal employment [6,7]. Other barriers to accessing public health facilities have been well-documented; these include documentation issues, fear of arrest, language and cultural differences, poor health literacy and many more [8]. To mitigate this issue, different stakeholders like the United Nations High Commissioner for Refugees (UNHCR), non-governmental organizations (NGOs), civil society organizations (CSOs) and private entities have established a wide range of health care services which are mostly free of charge or offered at a much lower price compared to that of public or private health facilities.

This positive development has been a huge step in facilitating thousands of RAS in Malaysia to access health care services without excessive out-of-pocket payment. To some extent, these services simultaneously address issues related to language barrier (eg, provision of interpreter), documentation (eg, accepting those without registration cards), and transportation (eg, free transportation). Still, there is a lot of room for improvement in these programs. In this paper, we would like to highlight a number of ‘deficits’ or impediments that may have limited the efficiency of existing initiatives and thus propose the need for a systematic mapping of health care services for RAS in this country. Our focus is mainly on non-state actors and service providers.

The first deficit is the tendency of some service providers (NGOs, CSOs, private entities, etc) to work in silos, without collaboration or coordination with other entities. While synchronization among stakeholders has definitely improved in recent years [9] – especially among the larger actors – coordination is relatively limited at several levels; among smaller NGOs, between smaller and larger ones, and across sectors. This has possibly resulted in the ‘replication’ of health programs and services, and work redundancy. Second, most health-related services tend to cluster at the acute/curative or primary care level. While these are highly needed, fewer programs are available at the promotive and preventive level, with much lesser initiatives in secondary or tertiary care. Similarly, it is not easy to find services that address specific needs – often unavailable in outpatient care settings – such as mental health, reproductive health, or dental care. For instance, a number of NGOs are known to provide mental health services for RAS, such as Medecins Sans Frontieres (MSF), MERCY Malaysia and Health Equity Initiatives (HEI) [10-12]. But it is unclear to what extent their coverage is or how adequate the services are. Third, some of these services are one-off in nature or seasonal, the most common reason being funding or budget constraint. Such lack of continuity leads to disruption of follow-ups and hampers the management of chronic diseases.

Other than that, most services tend to concentrate in Klang Valley – an urban conglomeration that includes Kuala Lumpur (KL) and several adjoining cities of Selangor. For instance, between 2004 and 2016, 70% of health programs conducted by MERCY Malaysia (one of the biggest NGOs that provides health services to marginalized populations in the country) that specifically targeted refugees, were in areas within Klang Valley [10]. In a similar vein, MSF had run most of its activities in Kuala Lumpur, Penang, Negeri Sembilan and Johor Bahru [11]. Even though these trends correspond with the pattern of RAS residence (77.1% are reported to live in Selangor, KL, Penang and Johor) [13], not much is known about the remaining RAS population that is scattered across six less developed states – Kedah, Terengganu, Pahang, Kelantan, Perak and Perlis – with regards to the availability and distribution of, and their access to, similar health care services. Lastly, there is rarely systematic data collection or data sharing among these service providers, a phenomenon that can be attributed to shortage of manpower and expertise, or other logistics issues.

**Figure:**
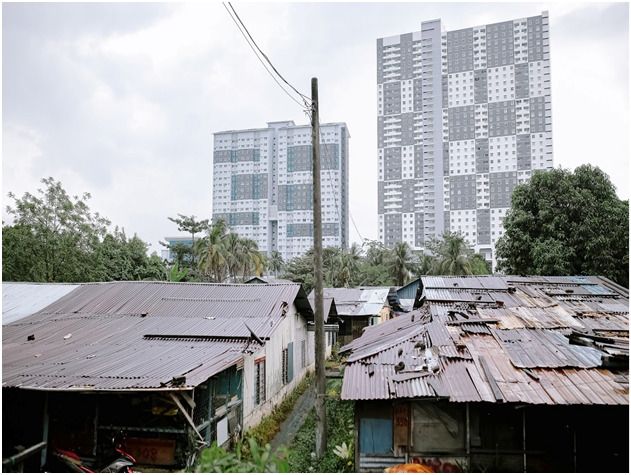
Photo: A slum area in Sentul, Kuala Lumpur, that shows the poor living condition of Rohingya refugees who mostly cannot access health care at government facilities. At the background, two posh condominium buildings are visible – an interesting image that implies inequality and health disparities (From the author’s own collection, used with permission).

Existing and future health care services for RAS in Malaysia can be greatly improved if current programs/initiatives are systematically mapped out. This exercise should aim at identifying all relevant non-state health actors/ service providers, the types of services being offered, the level and scope of service(s), the nature of programs (eg, static vs mobile) and where they cluster. The mapping of health care services for RAS will give a wide range of benefits. First, it can identify the gaps; which type(s) of service – at which level and where – are lacking. This knowledge can assist service providers in planning their future programs, (re)allocating health resources to where they are most needed, and enabling a more holistic approach in the provision of services for RAS. Second, mapping of health care services will pave the way for greater coordination between stakeholders (within and across sectors), by giving a clearer picture of who is doing what, and where. Readily available information in this regard will facilitate networking and more effective communication among service providers, resulting in better strategic planning, collaboration and mutual aid. This is crucial to drive efficiency, avoid wastage of resources or redundancy, and enhance the quality of services Most importantly, systematic plotting of existing health care services and programs will encourage methodical data collection and sharing – two fundamental aspects that can inform future programming and policies.

Needless to say, this ‘map’ is most impactful if it is dynamic, not static. Regular updating is key given the high mobility of RAS populations, the increasing complexity and changes in health needs, and the ambulatory nature of many of these services. Accordingly, the map can come in the form of conventional evidence map that is online and interactive or it may utilize the newer geo-mapping system. Last but not least, we would like to emphasize that this call for systematic mapping should not eclipse the more pressing need to provide a comprehensive socio-legal framework and to address the social determinants of health of RAS in Malaysia.
